# Supplementary material for: Patterns of Intron Gain and Loss in Fungi
Source: PLoS Biol. 2004 Nov 30;2(12):e422. doi: 10.1371/journal.pbio.0020422 (PMC532390; doi:10.1371/journal.pbio.0020422)
Supplement: Table S1 — Also available at http://genes.mit.edu/NielsenEtAl/. (4.3 MB ZIP). [file pbio.0020422.st001.zip › NielsenEtAl/html/543.html]

AN6255.1.NCU06741.1.MG01111.1.FG06268.1


```
 CLUSTAL W (1.82) Multiple Sequence Alignments - Introns Inserted


Sequence 1: NCU06741.1	84 aa
Sequence 2: MG01111.1	85 aa
Sequence 3: FG06268.1	62 aa
Sequence 4: AN6255.1	93 aa
Alignment Length: 93 aa
Number Identitical Residues: 45 aa
Alignment Score (without introns) 2596


MG01111.1 	-------MSDEERVTKPFKFVTG1-~VDARFPNTNQ~TKHCWQNYVDYHKCILAKGEDFA
NCU06741.1	-------MSDDERVTKPFKFVTG1-~VDARFPNVNQ~TKHCWQNYVDYHKCILAKGEDFA
FG06268.1 	-----------------------~-~-------MNQ~TKHCWQNYVDYHKCIIAKGEDFA
AN6255.1  	MGAIPEADPDEPVETKPFKFVTA1G1YDARFPQQNQ2TKHCWQNYVDYHKCVNAKGEDFR
          	 .: ..: ...   :.. .  :. .  .:  .. ** **************: ****** 

MG01111.1 	PCRQ0FWLAYRSLCPSSWYERWDSQRE1AGNFPVKLEQ
NCU06741.1	PCRQ0FWLAYRSLCPSGWYQRWDEQRE1AGNFPVKLE-
FG06268.1 	PCRQ0FWLSYRSLCPSGWYQRWDEQRE1SGNFPVKLDA
AN6255.1  	PCRQ0FYLAFRSLCPKPWTDRWDEQRE1AGNFPARLDR
          	**** *:*::*****. * :***.*** :****.:*:
```
